# From Reactive to Proactive: Using Preswarms to Prevent Harm in a Pediatric Cardiac Intensive Care Unit

**DOI:** 10.1097/pq9.0000000000000935

**Published:** 2026-10-21

**Authors:** L’Tanya J. Pierce-Thomas, Catherine A. Ferreira

**Affiliations:** From the Nemours Children’s Hospital, Cardiac Center, Wilmington, Del.

## Background:

Pediatric cardiac intensive care unit (CICU) patients are at high risk for healthcare-associated infections and conditions (HAI/HAC), including central line-associated bloodstream infection (CLABSI), catheter-associated urinary tract infection (CAUTI), and pressure injuries (PI). CICU patients have complex physiologies, multiple invasive devices, limited mobility, and prolonged intensive care unit stays. Because this population has predictable risk factors for CLABSI, CAUTI, and PI, we identified an opportunity to shift from reactive harm review to proactive prevention through structured risk identification and multidisciplinary collaboration.

## Objectives:

The objective was to reduce preventable HAI/HAC by proactively recognizing high-risk patients and initiating a preswarm focused on CLABSI, CAUTI, and PI prevention. A preswarm was defined as a multidisciplinary bedside huddle using a standardized process to assess overlapping harm risks, reinforce solutions for patient safety (SPS)-aligned prevention bundles, address barriers to bundle adherence, and empower frontline staff to act before serious harm occurred.

## Methods:

Patients triggered a preswarm based on individual harm risk or combined “triple threat” risk when overlapping CLABSI, CAUTI, and PI criteria were present (Fig. 1). Once identified, the Quality and Safety Coordinator and Cardiac Clinical Nurse Educator were notified to initiate the process. A structured chart review and bedside assessment evaluated patient history and trajectory, lines, drains, airways, devices, wounds, skin concerns, and adherence to SPS-aligned maintenance bundles. The multidisciplinary preswarm included the bedside nurse, provider team, respiratory therapy, wound care nursing, harm prevention and infection prevention partners, and other disciplines as indicated. Standardized prompts guided discussion of device necessity, offloading needs, bundle gaps, and prevention barriers. Each preswarm generated individualized prevention action items and a patient-specific plan with assigned ownership. The plan was documented in the medical record and displayed at the bedside to support shared mental models across caregivers and shifts. Patients were followed daily, and plans updated as clinical status and risk factors changed.

**Fig. 1.**
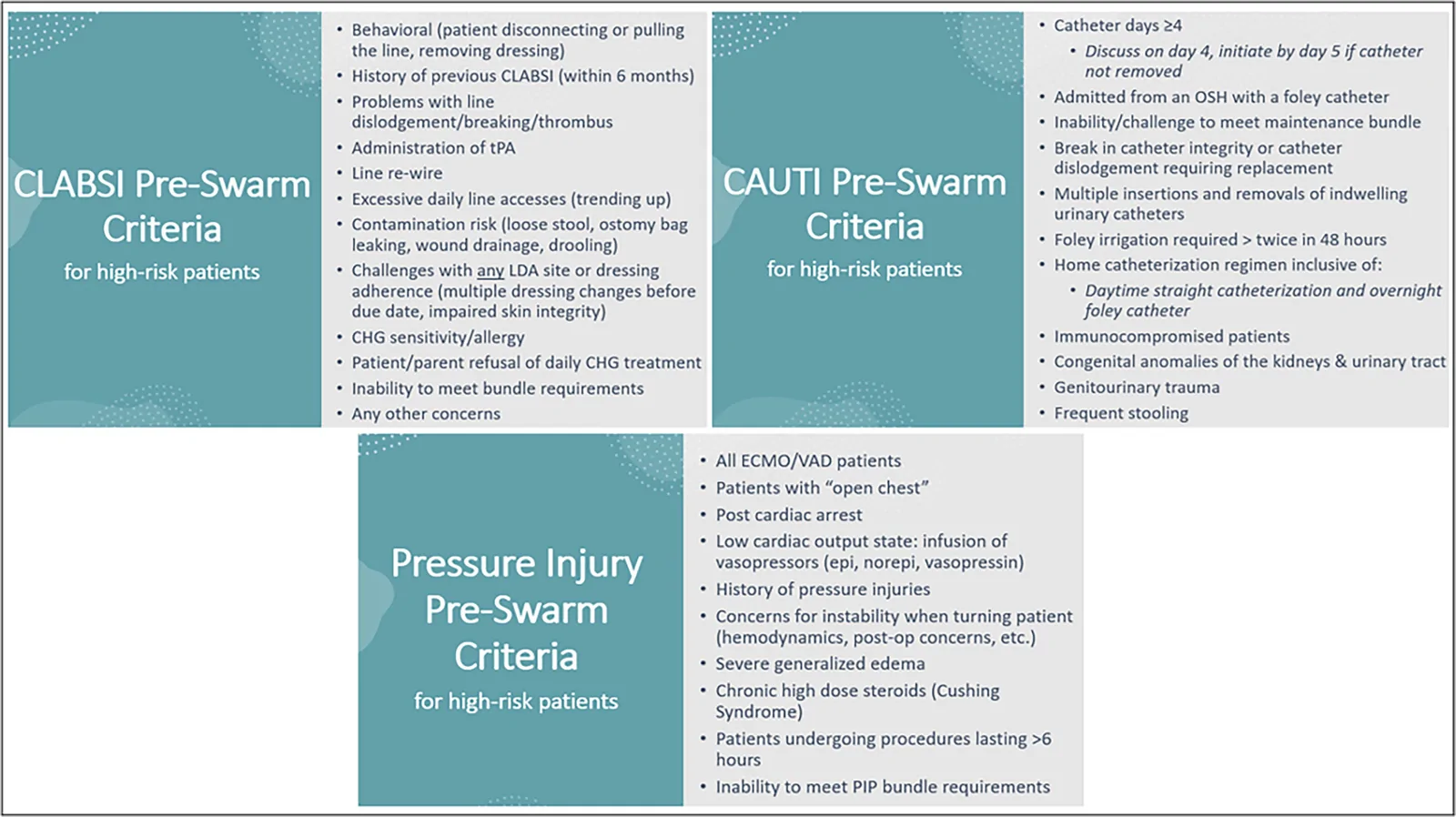
Preswarm criteria for CLABSI, CAUTI, and PI.

## Results:

In 2024, the CICU experienced preventable CLABSI, CAUTI, and PI, with most events device-related and none meeting the organizational definition of reportable. Although not reportable, these events highlighted the need for more reliable, proactive prevention. Following preswarm restructuring and standardization in 2025 with defined triggers, structured prework, multidisciplinary bedside huddles, and standardized documentation, the CICU demonstrated reductions in CLABSI, CAUTI, and PI (Fig. 2) CLABSI decreased from 2.33 with 7 events in 2024 to 0.44 with 1 event in 2025, an 81% reduction. CAUTI decreased from 3.82 with 2 events to 0, a 100% reduction. Overall PI decreased from 4.46 with 20 events to 0.67 with 2 events, an 85% reduction. Device-related PI decreased from 4.02 with 18 events to 0.67 with 2 events, an 83% reduction. Qualitatively, standardized preswarms improved risk recognition, shared situational awareness, prevention conversations, and barrier removal. Prevention plans were clearer across shifts, and bundle adherence became a multidisciplinary responsibility.

**Fig. 2.**
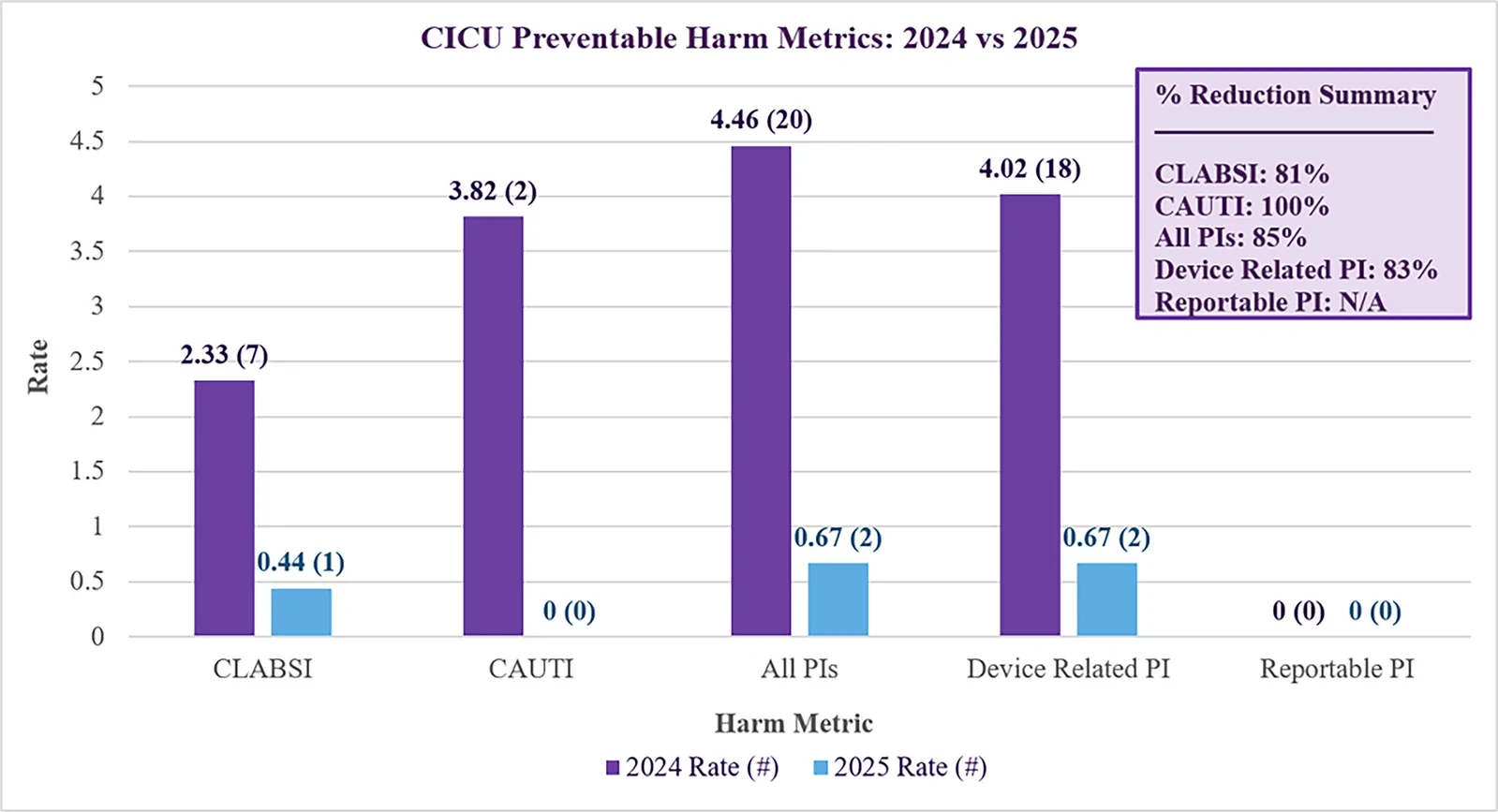
CICU Preventable Harm Metrics. Rates are per 1000 central line days, indwelling urinary catheter days and patient days, respectively.

## Conclusions/Implications:

Proactive multidisciplinary “triple threat” preswarms were associated with reduced HAI/HAC in high-acuity CICU patients. This SPS-aligned approach promotes early risk recognition, bundle adherence, barrier resolution, shared accountability, and safety culture, representing a scalable strategy to prevent serious harm.

## Acknowledgments

The authors acknowledge the multidisciplinary CICU team and harm prevention partners who contributed to this proactive prevention work.

